# Exploratory Analysis of Dental Age Differences in Children with Type 1 Diabetes Using the Demirjian Method

**DOI:** 10.3390/diagnostics16091395

**Published:** 2026-05-05

**Authors:** Maria Simona Dămășaru, Eugen Bud, Sorana Maria Bucur, Mariana Păcurar, Manuela Chibelean, Silvia-Izabella Pop, Alexandru Ștefan Zalana, Irina Elena Muntean, Lucian Cristian Petcu, Mariana Cornelia Tilinca

**Affiliations:** 1 Doctoral School, George Emil Palade University of Medicine, Pharmacy, Science, and Technology of Târgu Mureș, 38 Ghe. Marinescu Street, 540142 Târgu Mureș, Romania; damasaru.maria@gmail.com; 2 Department of Orthodontics, George Emil Palade University of Medicine, Pharmacy, Science, and Technology, 38 Ghe. Marinescu Street, 540139 Târgu Mureș, Romania; mariana.pacurar@umfst.ro (M.P.); manuela.chibelean@umfst.ro (M.C.); silvia.pop@umfst.ro (S.-I.P.); 3 Department of Dentistry, Faculty of Medicine, University “Dimitrie Cantemir” of Târgu Mureș, 540545 Târgu Mureș, Romania; 4 Faculty of Dentistry, Titu Maiorescu University, Gheorghe Petrașcu Street, 031593 Bucharest, Romania; alexandruzalana@gmail.com; 5 Algocalm SRL, 540360 Târgu Mureș, Romania; irinagauca@yahoo.com; 6 Scoala Doctorala de Medicina, Universitatea Ovidius Constanta, 900527 Constanța, Romania; lucian-cristian.petcu@365.univ-ovidius.ro; 7 Department of Internal Medicine I, Faculty of Medicine in English, George Emil Palade University of Medicine, Pharmacy, Science, and Technology of Târgu-Mureș, 540142 Târgu-Mureș, Romania; mariana.tilinca@umfst.ro

**Keywords:** dental age estimation, Demirjian method, Type 1 diabetes, biological maturation, orthodontic treatment timing, HbA1c

## Abstract

**Background:** Chronological age does not always accurately reflect biological maturation in children, particularly in the presence of systemic conditions. Dental age is widely used as a biological maturity indicator; however, the impact of Type 1 diabetes mellitus on dental development remains unclear and inconsistently reported. Objective: This paper aims to explore differences between dental age (DA) and chronological age (CA) in children with Type 1 diabetes compared to healthy controls, and to assess the association between glycemic control (HbA1c) and dental maturation. **Materials and Methods**: This observational comparative study included 90 children aged 8–15 years: 45 with Type 1 diabetes and 45 age- and sex-matched healthy controls. Dental age was estimated using the Demirjian method and compared with chronological age. Group comparisons were performed using independent *t*-tests, while paired *t*-tests assessed within-group differences. Linear regression analysis evaluated the association between HbA1c and dental age. Effect sizes and 95% confidence intervals were reported. **Results:** In the diabetes group, dental age was significantly higher than chronological age (mean difference = 1.56 years, *p* < 0.001), indicating advanced dental maturation. No significant difference between dental and chronological age was observed in the control group. Dental age was also significantly higher in the diabetes group compared to controls (mean difference = 1.61 years, *p* < 0.001; Cohen’s d = 0.93). HbA1c levels were positively associated with dental age (R^2^ = 0.409, *p* < 0.01), suggesting that metabolic control may contribute to variability in dental maturation. **Conclusions:** Children with Type 1 diabetes appear to exhibit advanced dental maturation compared to healthy peers. Glycemic control may be associated with this variation, although the findings should be interpreted within the exploratory framework of the study. Dental age assessment should be used cautiously and in conjunction with other maturity indicators, particularly in children with systemic conditions.

## 1. Introduction

Accurate assessment of growth and maturation in children and adolescents is essential for clinical decision-making across dentistry, orthodontics, pediatrics, and forensic science [1,2]. Although chronological age (CA) remains the most commonly used reference, it often fails to reflect true biological development due to substantial inter-individual variability influenced by genetic, environmental, nutritional, and systemic factors [3,4]. Furthermore, growth is not a uniform process; distinct biological systems—skeletal, dental, somatic, and sexual—follow different developmental trajectories, as demonstrated by classical growth models [5,6,7]. As a result, reliance on chronological age alone may lead to inaccuracies in diagnosis and suboptimal timing of therapeutic interventions, particularly in growth-dependent disciplines such as orthodontics [8].

To overcome these limitations, biological maturity indicators have been increasingly adopted. Among them, dental age (DA) is considered a reliable and reproducible marker of developmental status [9,10]. Dental maturation, assessed through tooth mineralization, follows a continuous and genetically regulated process that is comparatively less susceptible to environmental fluctuations than other maturity indicators [11]. This relative stability makes dental age particularly valuable in children affected by systemic conditions, where other indicators of growth may be altered or less reliable [11,12].

Dental age assessment has broad applications in both clinical and scientific contexts. In pediatric dentistry and orthodontics, it contributes to treatment planning and timing by identifying key developmental stages [2,13,14,15,16,17]. In pediatrics and endocrinology, it provides complementary information for evaluating growth patterns and systemic disorders [18,19,20]. In forensic science, dental age plays a crucial role in estimating the age of unidentified individuals or minors in legal contexts, while in anthropology and archaeology, it assists in reconstructing growth patterns in past populations [21,22,23,24,25,26].

Radiographic evaluation of tooth development has become the preferred approach for estimating dental age, given the limitations of eruption-based methods, which are highly influenced by local and environmental factors [27,28,29,30]. Among the various techniques available, the method proposed by Demirjian, Goldstein, and Tanner remains one of the most widely used and validated worldwide [1,10,31,32,33,34,35]. Its popularity stems from its reproducibility, standardized scoring system, and applicability across diverse populations [33,34,35,36]. However, despite its methodological robustness, dental age estimation does not always correlate with other indicators of biological maturation, reflecting the complex and partially independent nature of developmental processes [37,38,39,40].

Systemic diseases have the potential to alter the tempo of biological maturation, including dental development [18,41,42]. Type 1 diabetes mellitus (juvenile form), characterized by chronic metabolic dysregulation and worsening the life quality overall, has been associated with changes in growth patterns, mineral metabolism, and craniofacial development [43,44,45,46,47,48]. Nevertheless, the impact of this condition on dental maturation remains insufficiently clarified, with studies reporting inconsistent findings ranging from delayed to accelerated or unchanged dental development relative to chronological age [43,44,45,46]. These discrepancies may reflect differences in study design, population characteristics, and methodological approaches.

Given the clinical and forensic importance of accurate age estimation, clarifying the relationship between dental and chronological age in children with systemic conditions is essential. In particular, understanding whether metabolic control influences dental maturation may have implications for treatment planning and age assessment in both clinical and medico-legal settings.

Therefore, the present study aimed to explore differences between dental age, estimated using the Demirjian method, and chronological age in children with Type 1 diabetes compared to age- and sex-matched healthy controls, and to evaluate the potential association between glycemic control (HbA1c) and dental maturation.

## 2. Materials and Methods

### 2.1. Study Design and Ethical Approval

This observational comparative study investigated the relationship between dental age (DA) and chronological age (CA) in children with Type 1 diabetes mellitus and evaluated the potential association between glycemic control and dental maturation.

The study protocol was approved by the Ethics Committee of the “George Emil Palade” University of Medicine, Pharmacy, Science, and Technology of Târgu Mureș (Decision No. 3787/19.05.2025) and conducted in accordance with the principles of the Declaration of Helsinki. Written informed consent was obtained from all participants and their legal guardians before inclusion.

### 2.2. Study Population

A total of 90 children aged between 8 and 15 years were included and allocated into two groups.

- Diabetes group (DG): 45 children diagnosed with Type 1 diabetes mellitus and undergoing regular endocrinological follow-up.
- Control group (CG): 45 systemically healthy children matched for age, sex, and dento-maxillary characteristics.

To minimize potential confounding related to craniofacial growth patterns, all participants presented Angle Class I dento-maxillary relationships.

### 2.3. Inclusion and Exclusion Criteria

*Inclusion criteria*

Participants were eligible if they met the following criteria:

- Age between 8 and 15 years;
- Availability of high-quality panoramic radiographs;
- Presence of all seven permanent mandibular teeth required for dental age assessment;
- Diagnosis of Type 1 diabetes mellitus (for the DG);
- Absence of systemic disease (for the CG);
- Angle Class I occlusal relationship.

*Exclusion criteria*

Participants were excluded if they had:

- Systemic diseases affecting growth or mineral metabolism;
- Craniofacial anomalies or syndromes;
- History of orthodontic treatment;
- Congenital absence of mandibular teeth required for analysis;
- Radiographs of insufficient quality for accurate assessment.

### 2.4. Assessment of Glycemic Control

In the Diabetes group, glycemic control was assessed using glycated hemoglobin (HbA1c) values retrieved from medical records corresponding to the time closest to radiographic examination.

HbA1c reflects mean blood glucose levels over the preceding 2–3 months and was analyzed both as a continuous variable and as a categorical variable:

- Good metabolic control: HbA1c < 7.5%;
- Suboptimal metabolic control: HbA1c ≥ 7.5%.

Data regarding disease duration, insulin therapy, pubertal status, and nutritional indicators such as body mass index (BMI) were not consistently available and were therefore not included in the analysis. These variables represent important biological and clinical confounders that may influence dental maturation.

### 2.5. Dental Age Assessment

Dental age was estimated using the Demirjian method, a radiographic technique based on the evaluation of tooth calcification stages. Panoramic radiographs were used to assess the seven left permanent mandibular teeth, excluding the third molar [10,12,33,34,36,37,38,39].

Each tooth was assigned a developmental stage (A–H) according to standardized morphological criteria describing crown and root formation. These stages represent successive phases of dental mineralization, from initial cusp formation to complete root apex closure.

Each stage was converted into a sex-specific maturity score, and the individual scores were summed to obtain a total maturity score. This score was subsequently transformed into dental age using standardized reference tables.

In cases where a tooth could not be evaluated on the left side, the corresponding contralateral tooth was assessed.

All radiographic evaluations were performed by a trained and calibrated examiner following standardized Demirjian criteria. Calibration was achieved through repeated assessments on a subset of radiographs before the main analysis.

However, formal assessment of intra- and inter-examiner reliability using statistical measures such as the intraclass correlation coefficient (ICC) or Cohen’s kappa was not performed. Therefore, the reproducibility of the measurements cannot be quantitatively confirmed.

### 2.6. Chronological Age Determination

Chronological age was calculated by subtracting the date of birth from the date of radiographic examination and expressed in decimal years.

The difference between dental age and chronological age (DA − CA) was computed for each participant to assess the direction and magnitude of dental maturation. Positive values indicated advanced maturation, while negative values indicated delayed development.

### 2.7. Statistical Analysis

Statistical analyses were performed using appropriate parametric methods following confirmation of normal data distribution (Shapiro–Wilk test) and homogeneity of variances (Levene’s test).

- Independent samples *t*-tests were used to compare CA, DA, and HbA1c between groups.
- Paired samples *t*-tests were used to compare CA and DA within each group.
- Lin’s concordance correlation coefficient was applied to assess agreement between CA and DA.
- Linear regression analysis was performed to evaluate the association between HbA1c and dental age in the Diabetes group.

Effect sizes (Cohen’s d) and 95% confidence intervals were calculated for all major comparisons. Post hoc statistical power was estimated using G*Power (version 3.1.9.7; Heinrich Heine University Düsseldorf, Düsseldorf, Germany). A *p*-value < 0.05 was considered statistically significant. To account for the potential confounding effect of chronological age, an additional linear regression analysis was performed using dental age advancement (DA − CA) as the dependent variable and HbA1c as the independent variable.

## 3. Results

Descriptive statistics for chronological age (CA) and dental age (DA) in both groups are presented in Table 1, while HbA1c values for the Diabetes group are summarized in Table 2.

The two groups were comparable in terms of chronological age. In the Diabetes group, Lin’s concordance correlation coefficient (ρc = 0.693; 95% CI: 0.591–0.773) indicated only moderate agreement between chronological age and dental age. Although the Pearson correlation coefficient was extremely high (ρ = 0.998), reflecting a strong linear relationship (precision), the substantially lower concordance coefficient indicates the presence of systematic bias between the two measurements (lack of accuracy). This suggests that, despite the variables changing in parallel, dental age consistently deviates from chronological age in this group.

Baseline comparisons between groups are detailed in Table 3. No significant difference was observed for chronological age (*p* = 0.757), confirming appropriate matching. In contrast, dental age was significantly higher in the Diabetes group (*p* < 0.001), with a mean difference of 1.61 years. HbA1c levels were also significantly higher in the Diabetes group (*p* < 0.001).

Before inferential analysis, assumptions for parametric testing were verified. The Shapiro–Wilk test indicated normal distribution of variables, and Levene’s test confirmed homogeneity of variances (all *p* > 0.05).

### 3.1. Agreement Between Dental and Chronological Age

The agreement between dental age and chronological age was assessed using Lin’s concordance correlation coefficient.

In the Diabetes group, concordance was moderate (ρc = 0.693; 95% CI: 0.591–0.773). Although precision was high (ρ = 0.998), the lower bias correction factor (Cb = 0.694) indicated a systematic overestimation of dental age relative to chronological age.

In contrast, the Control group demonstrated near-perfect agreement (ρc = 0.998; 95% CI: 0.996–0.999), with both high precision and accuracy (ρ = 0.999; Cb = 0.999), indicating strong concordance between the two measures in healthy children.

### 3.2. Between-Group and Within-Group Comparisons

Between-group comparisons

There was no significant difference in chronological age between the Diabetes and Control groups (*p* = 0.757).

However, dental age was significantly higher in the Diabetes group (12.62 ± 1.76 years) compared to the Control group (11.01 ± 1.68 years) (*p* < 0.001), with a large effect size (Cohen’s d = 0.93), indicating a clinically meaningful difference in dental maturation.

HbA1c levels were also significantly higher in the Diabetes group (*p* < 0.001), confirming the expected metabolic distinction between groups.

Within-group comparisons

Within-group analysis revealed distinct patterns. In the Diabetes group, dental age was significantly higher than chronological age (mean difference = 1.56 years, *p* < 0.001), indicating advanced dental maturation.

In contrast, no significant difference between dental and chronological age was observed in the Control group (mean difference = 0.06 years, *p* = 0.865), supporting concordance between these measures in healthy children.

### 3.3. Association Between Glycemic Control and Dental Age

The relationship between glycemic control and dental maturation was evaluated using linear regression analysis in the Diabetes group.

A significant positive association was identified between HbA1c and dental age (*p* < 0.001), with HbA1c explaining 40.9% of the variance in dental age (R^2^ = 0.409). The standardized regression coefficient (β = 0.640) indicated a moderate-to-strong relationship.

An increase of 1% in HbA1c was associated with an estimated increase of approximately 2.58 years in dental age (B = 2.577, SE = 0.472, *p* = 0.001). However, although statistically significant, this effect size should be interpreted with caution, as it may reflect model instability related to the limited sample size and the narrow variability of HbA1c values rather than a true biological magnitude of effect.

### 3.4. Graphical Representation of Findings

**Figure 1:**
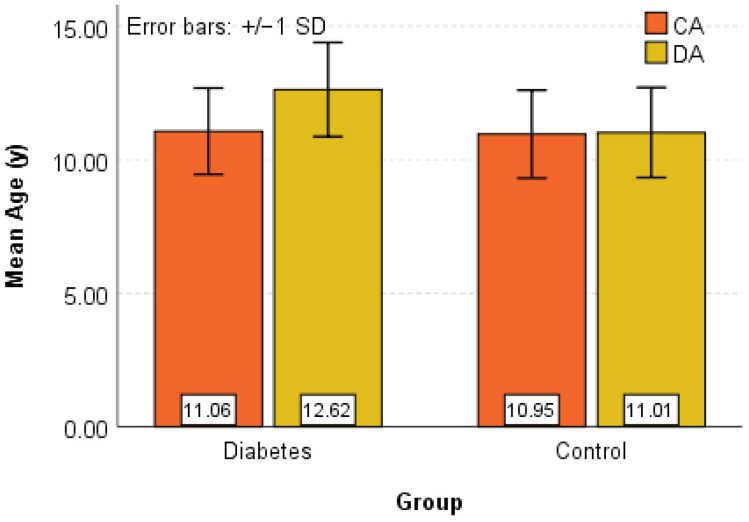
Box-Plot and Error-Bar graphical representation for CA (y) and DA (y) in the Control and Diabetes groups.

**Figure 2:**
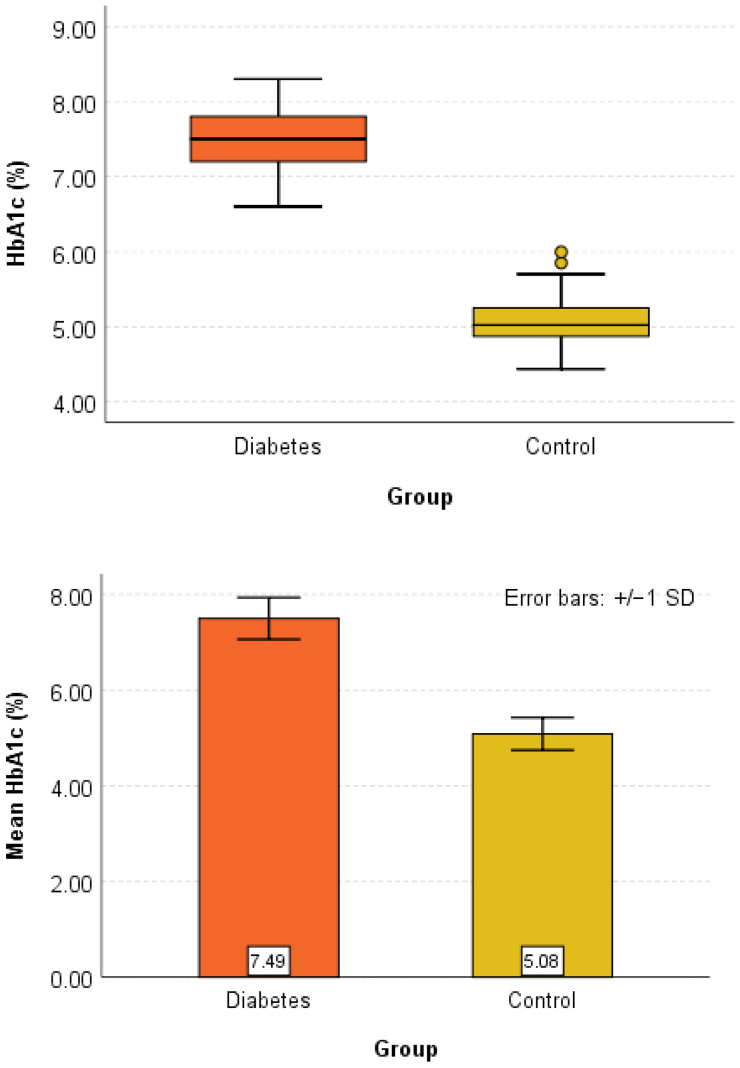
Box-Plot and Error-Bar graphical representation for HbA1c (%) in the Control and Diabetes groups.

**Figure 3:**
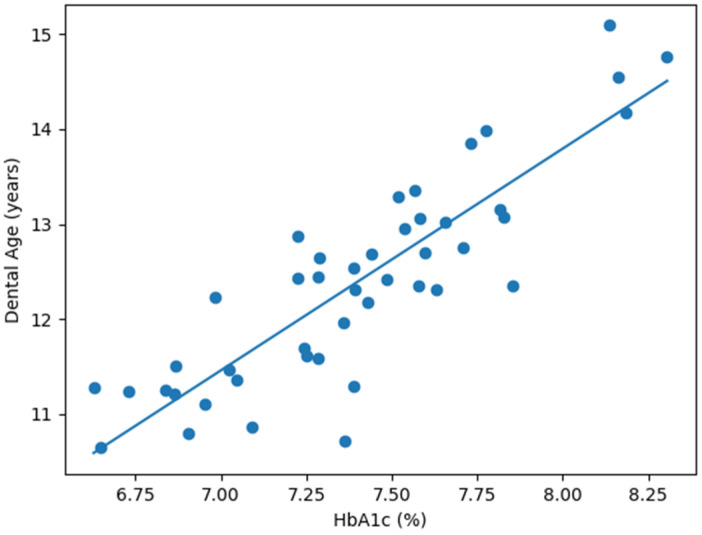
Scatter plot representation of DA as a function of HbA1c for the Diabetes group.

Graphical analyses supported the statistical results and are presented in Figure 1, Figure 2 and Figure 3.

Figure 1 illustrates the distribution of chronological and dental age across both groups using box plots and error bars, highlighting the higher dental age values observed in the Diabetes group.

Figure 2 presents the distribution of HbA1c values, clearly showing higher levels in the Diabetes group compared to controls.

Figure 3 depicts the relationship between dental age and HbA1c in the Diabetes group, demonstrating a positive linear trend consistent with the regression analysis.

## 4. Discussion

Within the exploratory framework of the present study, children with Type 1 diabetes mellitus demonstrated significantly advanced dental maturation compared with age- and sex-matched healthy controls. In the Diabetes group, dental age exceeded chronological age by a mean of 1.56 years, whereas no significant discrepancy was observed in the Control group. In addition, HbA1c levels showed a significant positive association with dental age, suggesting that metabolic control may contribute to variability in the tempo of dental development.

These findings support the hypothesis that systemic metabolic disturbances associated with Type 1 diabetes may influence odontogenesis and the pace of dental maturation [43,45,47]. Although dental development is generally considered a relatively stable biological process, less susceptible to short-term environmental influences than skeletal or somatic growth [2,12,49], the present results suggest that chronic endocrine and metabolic imbalance may alter this trajectory.

A key distinction should be emphasized between accelerated dental maturation and accelerated tooth eruption, as these processes are biologically and clinically distinct. The present study assessed calcification-based dental development using the Demirjian method rather than eruption status. While previous studies have reported earlier tooth eruption in children with diabetes [43,44,45], eruption may be affected by local factors such as space conditions, occlusal forces, or periodontal status [27,28]. By contrast, the present findings specifically reflect an apparent acceleration in the mineralization and developmental stages of permanent teeth.

The discrepancy between the very high Pearson correlation and the lower concordance coefficient highlights that correlation alone does not imply agreement, as systematic bias may still be present.

Importantly, when accounting for chronological age using dental age advancement (DA−CA), the association between HbA1c and dental maturation remained statistically significant. This suggests that the relationship is not solely explained by age-related effects and may reflect a potential independent influence of glycemic control on dental development. Notably, the magnitude of the association was substantially lower than in the unadjusted model, indicating that the initial estimate may have been inflated by age-related confounding. From a biological perspective, several mechanisms may contribute to these findings. Insulin and insulin-like growth factors (IGFs) are known to regulate cellular proliferation, differentiation, and extracellular matrix formation in mineralized tissues [44,45,47]. Experimental evidence suggests that IGF-mediated pathways may influence odontoblast function, dentinogenesis, and mineral deposition, potentially accelerating calcification processes. In addition, disturbances in calcium-phosphate metabolism, frequently observed in diabetic patients, may further modulate dental mineralization.

The present findings are consistent with the concept that dental maturation may progress independently of other developmental systems. Previous studies have reported inconsistent relationships between dental, skeletal, and chronological age [8,50,51]. While others found weak or absent correlations [52,53,54], some authors described strong concordance between dental and skeletal maturity [55,56,57,58,59]. These discrepancies suggest that dental development, although influenced by systemic hormonal factors, may retain a degree of biological autonomy.

Clinically, these findings are relevant for pediatric dentistry and orthodontics. Accurate assessment of developmental status is essential for timing growth-dependent interventions, particularly in orthodontic treatment planning. If dental age is advanced in children with Type 1 diabetes, reliance on chronological age alone may lead to suboptimal therapeutic timing. Therefore, dental maturation should be interpreted alongside skeletal and chronological indicators, particularly in children with systemic metabolic conditions.

The results also have potential forensic implications. Dental age estimation is frequently used in age assessment of minors and unidentified individuals [21,22,23,24,25,37]. If systemic conditions such as Type 1 diabetes are associated with accelerated dental maturation, age may be overestimated when dental methods are used in isolation. This may be particularly relevant in medico-legal settings involving age thresholds.

Several limitations should be acknowledged. First, no a priori sample size calculation was performed, and although post hoc analysis suggested adequate statistical power, this remains a methodological limitation. In addition, the relatively small sample size limits the generalizability of the findings. Third, significant biological and clinical confounders, including duration of diabetes, pubertal stage, nutritional status (e.g., BMI), and socioeconomic factors, were not accounted for in the analysis. These variables may influence growth and maturation processes and could have affected the observed association between HbA1c and dental age. Furthermore, formal intra- and inter-observer reliability was not quantified using statistical measures such as ICC or Cohen’s kappa, which represents a methodological limitation, as dental age estimation is inherently observer-dependent.

Future multicenter studies with larger cohorts and longitudinal follow-up are warranted to confirm these findings and better clarify the role of metabolic control in dental development.

## 5. Conclusions

Within the exploratory framework of this study, children with Type 1 diabetes mellitus appear to exhibit advanced dental maturation compared with healthy peers, as reflected by higher dental age relative to chronological age.

Glycemic control, as assessed by HbA1c, was significantly associated with dental age advancement, suggesting a potential independent relationship with dental maturation. However, given the exploratory design and sample size, these findings should still be interpreted with caution. These findings highlight that dental maturation may be influenced by systemic metabolic conditions and may not always correspond closely with chronological age. From a clinical and forensic perspective, dental age should therefore be interpreted in conjunction with other maturity indicators, particularly in children with systemic diseases.

Further longitudinal and multicenter studies are required to confirm these observations and to better understand the mechanisms underlying the relationship between metabolic control and dental development.

## Figures and Tables

**Table 1:** Descriptive statistics for CA (y) și DA (y).

		N	Mean	Median	SD	Range	Min	Max	P25	P75	IQR
Diabetes	CA (y)	45	11.058	11.100	1.615	5.500	8.400	13.900	9.650	12.450	2.800
	DA (y)	45	12.618	12.600	1.762	6.100	9.600	15.700	11.100	14.150	3.050
Control	CA (y)	45	10.951	10.900	1.648	5.400	8.400	13.800	9.450	12.350	2.900
	DA (y)	45	11.011	11.000	1.683	5.700	8.300	14.000	9.450	12.350	2.900

**Table 2:** Descriptive statistics for HbA1c (%).

		N	Mean	Median	SD	Range	Min	Max	P25	P75	IQR
HbA1c (%)	Diabetes	45	7.491	7.500	0.437	1.700	6.600	8.300	7.200	7.800	0.600

**Table 3:** Baseline characteristics of the study population.

Variable	Diabetes Group (n = 45)	Control Group (n = 45)	*p*-Value
Age (years), mean ± SD	11.06 ± 1.62	10.95 ± 1.65	0.757
Dental Age (years), mean ± SD	12.62 ± 1.76	11.01 ± 1.68	<0.001
HbA1c (%), mean ± SD	7.49 ± 0.44	5.08 ± 0.34	<0.001

## Data Availability

The original contributions presented in this study are included in the article. Further inquiries can be directed to the corresponding authors.
